# Odorant Receptor C-Terminal Motifs in Divergent Insect Species

**DOI:** 10.1673/031.008.5301

**Published:** 2008-09-22

**Authors:** Raymond Miller, Zhijian Tu

**Affiliations:** ^1^ Department of Biochemistry, Virginia Polytechnic Institute and State University, Blacksburg, VA 24061

**Keywords:** mosquito, G-protein coupled receptor, MEME, heterodimer, hidden markov model, Or83b

## Abstract

Insect odorant receptors are a large family of seven transmembrane proteins believed to be G-protein coupled receptors. The peptide sequences of two odorant receptors within a given species may share as little as 17% identity, and there is limited similarity between receptors of divergent species. One exception is DmOr83b, which is found in *Drosophila melanogaster* and is highly conserved in at least ten other insect species. DmOr83b is broadly expressed in most of the olfactory sensory neurons of *D. melanogaster* at most developmental stages, while other odorant receptors tend to have more restricted and specific expression patterns. DmOr83b is critical for *D. melanogaster* olfaction, and it is involved in properly localizing other odorant receptors possibly by forming heterodimers with these receptors. The C-terminal region has been implicated as sites for such heterodimer formation. Multiple em for motif elicitation (MEME), a hidden markov model based program, was used to uncover three conserved motifs in the C-termini of a vast majority of the odorant receptor peptides from *Anopheles gambiae*, *D. melanogaster*, and *Apis mellifera*. These motifs are also found in DmOr83b and its orthologs and the order of these motifs is conserved as well. The conservation of these motifs among divergent odorant receptors in divergent species suggests functional importance. We propose that these motifs are involved in receptor- receptor protein interactions, contributing to the heterodimer formation between DmOr83b (or its orthologs) and other odorant receptors.

## Introduction

Insect olfaction and olfactory signaling is a rapidly growing area of research (Rutzler and Zwiebel 2005). Several protein families are being studied that include odorant binding proteins, sensory neuron membrane proteins, odorant degrading enzymes, and odorant receptors. A large body of recent literature has been written on insect odorant receptors (Clyne et al. 1999; Vosshall et al. 1999; Hill et al. 2002; Sakurai et al. 2004; Robertson and Wanner 2006). Most of the papers addressing insect odorant receptors report either the discovery of receptor genes in an insect species (Hill et al. 2002; Robertson et al. 2003), and/or the expression of selected odorant receptor genes at various points of the organism's life cycle (Melo et al. 2004). Odorant receptor gene expression is usually localized to the insect sensory organs such as antenna and maxillary palp (Vosshall et al. 2000; Fox et al. 2001), and more recently have been found to be expressed in the proboscis (Kwon et al. 2006). There are also a growing number of papers addressing the specific functions of several odorant receptor proteins (Wetzel et al. 2001; Hallem et al. 2004; Larsson et al. 2004; Sakurai et al. 2004).

Insect odorant receptors have been reported to be putative G-protein coupled receptors (Clyne et al. 1999; Gao and Chess 1999; Hill et al. 2002), but recently this status has been questioned (Benton 2006; Wistrand et al. 2006). The most extensively researched insect odorant receptor is DmOr83b in *Drosophila melanogaster*. A highly conserved ortholog of DmOr83b has been found in all insect species with sufficient genomic sequence information. This list includes *D. melanogaster* (Vosshall et al. 2000), *Anopheles gambiae* (Pitts et al. 2004), *An. stephensi* (R. Miller and Z. Tu, unpublished data), *An. quadriannulatus* (R. Miller and Z. Tu, unpublished data), *Aedes aegypti* (Melo et al. 2004), *Culex quinquefaciatus* (Xia and Zwiebel 2006), *Bombyx mori* (Sakurai et al. 2004), *Heliothis virescens* (Krieger et al. 2002), *Apis mellifera* (Robertson and Wanner 2006), and *Tribolium castaneum* (GenBank Accession XP_973196. Note that the GenBank name for OR is GPROR. This is in contrast to the vast majority of the other insect odorant receptors, which are not conserved between species of different genera. DmOr83b is broadly expressed in most of the olfactory sensory neurons of *D. melanogaster* at most stages of development (Vosshall et al. 2000; Larsson et al. 2004). This again is in contrast to other odorant receptors, which have been reported to have a restrictive expression pattern (Vosshall et al. 2000; Fox et al. 2001). *D. melanogaster* lacking a copy of DmOr83b are not able to respond to olfactory cues, and other odorant receptors are not properly localized to the membrane of olfactory sensory neurons (Larsson et al. 2004). DmOr83b is capable of forming a heterodimer with at least one *D. melanogaster* odorant receptor: DmOr43a (Neuhaus et al. 2005). The requirement of a heterodimer of two G-protein coupled receptors has only been previously observed in the GABA complex where heterodimer formation is required for the function of potassium/calcium channels (Jones et al. 1998; White et al. 1998). Benton and co-authors provides further evidence of heterodimer formation involving DmOr83b with DmOr22a/b, and additionally point to the C-terminal domain of odorant receptor peptides as being the site of heterodimer formation (Benton et al. 2006). The specific location(s) of the protein-protein interaction(s) were not explored. However, previous reports have indicated limited amino acid conservation occurring in the C-terminal end of *D. melanogaster* odorant receptor (DmOr) peptides, including a nearly invariable tryptophan residue (Clyne et al. 1999; Scott et al. 2001; Vosshall 2003).

Using a hidden markov model based program called multiple em for motif elicitation (MEME) (Bailey and Elkan 1994), we have discovered three C-terminal motifs in 76 of the 79 previously annotated *An. gambiae* odorant receptor peptides (Hill et al. 2002). Subsequent analysis indicates that these motifs are conserved within the odorant receptor peptides of *D. melanogaster* and *Ap. mellifera* (Robertson and Wanner 2006). This is significant given that it has been reported that insect odorant receptor peptides are highly divergent within and between species (Clyne et al. 1999; Vosshall et al. 1999; Hill et al. 2002; Vosshall 2003). For example, the amino acid identity between insect odorant receptors of the same species is only 17% in some cases (Vosshall 2003). We hypothesize that these motifs are protein-protein interaction sites involved in odorant receptor-odorant receptor interactions or potentially heterodimer formation between DmOr83b and other odorant receptors.

## Materials and Methods

### Alignment of *An. gambiae* odorant receptor peptides with ClustalW

All 79 *An. gambiae* odorant receptor peptides (Hill et al. 2002) were aligned using ClustalW v 1.83.1 (Thompson et al. 1994). Default parameters were used (multiple alignment gap opening penalty = 10, gap extension penalty = 0.2). Alignments were illustrated using the Jalview Java alignment editor (Clamp et al. 2004).

### Motif discovery in odorant receptors peptides using MEME

*An. gambiae* and *Ap. mellifera* odorant receptor peptide sequences were obtained from the supplementary material of two separate studies (Hill et al. 2002; Robertson and Wanner 2006). Fifty-nine *D. melanogaster* odorant receptor peptides were obtained from the Ensembl database (http://www.ensembl.org) and were used in the analysis. The program multiple em for motif elicitation (MEME) (Bailey and Elkan 1994) (http://meme.sdsc.edu/meme/) version 3.5.1 was compiled on a Macintosh computer running Mac OS 10.4.8. Each MEME analysis was run with peptide dataset from each species as input. For all three datasets MEME was run using the following command line: *meme dataset_name -protein -mod zoops -minw 15 maxw 45 -wg 8 -ws 0.2 -evt .00001 -nmotifs 8*. The program command call is *meme*, while *dataset_name* identifies the input dataset, -*protein* indicates the dataset contained peptide sequences, and -*mod* defines the search model. The remaining parameters were -*minw*, which sets the minimum possible motif width at 15 residues, -*maxw*, which sets the maximum possible motif width at 45 residues, -*wg*, which is the gap opening penalty, -*ws*, which is the gap extension penalty, -*evt*, which is the maximum e-value for a motif to be reported, and -*nmotifs*, which indicated the number of motifs that are searched for in the input dataset. Gap opening and extension penalties were reduced from the default values of *wg*=11 and *ws*=1 to *wg*=8 and *ws*=0.2 to reduce artificial breakup of the motifs due to small insertions or deletions. In addition to searching for the top 3 motifs as set by default, -*nmotifs* 8 was used to determine whether more than three motifs existed in each dataset.

### MAST searching of *An. gambiae* gustatory receptor peptides for odorant receptor motifs

The motif alignment and search tool (MAST) (Bailey and Gribskov 1998), another program in the MEME package, was used to search for AgOr motifs in all 76 *An. gambiae* GRs (Hill et al. 2002). MAST version 3.5.1 was installed as part of the MEME package (see above). Command line used for MAST was: *mast motif_matricies_found_by_meme -d database_of_AgGrs*. The *motif_matricies_found_by_meme* are the profile matrices of the motifs found in a previous MEME analysis and they effectively define the motifs. These matrices were used to search the *database_of_AgGrs*, where AgGrs stands for *An. gambiae* gustatory receptors. No other parameters were used.

### Weblogo diagrams

All weblogo diagrams were constructed using the weblogo program (Crooks et al. 2004) (http://weblogo.berkeley.edu/). MEME output includes BLOCKS of the motifs. If an odorant receptor peptide sequence was found to have a motif, the part of the peptide sequence that contains that motif was used in an alignment, which produced an aligned BLOCK. The aligned BLOCK was used to construct weblogos.

## Results

### ClustalW alignments of *An. gambiae* odorant receptors

An alignment of all 79 *An. gambiae* odorant receptor (AgOr) peptides using the multiple sequence alignment program ClustalW (Thompson et al. 1994) revealed very little strict sequence conservation (Figure 1). There were a small number of conserved or highly prevalent residues located in the C-terminal region (Figure 1, blue-colored residues). One of these highly conserved residues is a tryptophan residue found in all but four AgORs. The lack of strict sequence conservation in AgOrs, and the prevalence of the conserved tryptophan residue is consistent with what has been previously reported for DmOr peptides (Vosshall 2003).

### MEME identifies c-termlnal motifs in *An. gambiae* odorant receptors

To locate conserved patterns a hidden markov model based program named multiple em for motif elicitation (MEME) was used (Bailey and Elkan 1994). MEME has been used to locate potential regulatory sites in sequences upstream of genes (Ohler et al. 2002), potential proteinprotein interaction domains (Fang et al. 2005), and homologous genes missed by homology search Janssen et al. 2004). One key advantage of MEME over common alignment programs is its ability to find motifs that are not absolutely conserved in consensus sequence. Other advantages of MEME are its speed, no need for prior knowledge about a dataset, and its ability to locate motifs that may not be in the same order through all members of a dataset.

**Figure 2.**
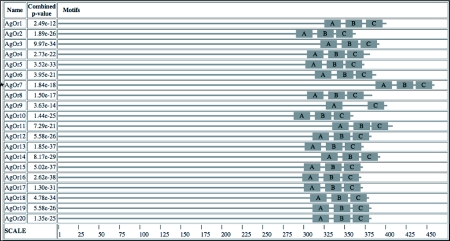
C-terminal motifs found in *Anopheles gambiae* odorant receptors. The image was taken directly from the MEME output and shows the position of three C-terminal motifs located in the first 20 AgOr peptides. Only the first 20 AgOrs were shown to save space. The asterisk points to AgOr7, which is the *An. gambiae* ortholog of DmOr83b. The combined p-value is the probability of finding a match of a sequence in this dataset to a group of motifs by random chance.

All 79 AgOr peptides were used as input for MEME run using a gap opening penalty of eight and a gap extension parameter of 0.2. Three motifs were identified within the dataset all with highly significant e-values (4.2e^-401^, 4.5e^-367^, and 1.1e^-332^) (Figure 2, Table 1). All three motifs were present within the last 70 or 90 amino acid residues of the C-terminal end of AgOr peptides, and 76 out of 79 (96%) AgOr peptides had all three motifs. The order of the motifs from N-terminal to C-terminal is motif 3, motif 2, and motif 1. MEME numbers the motifs according to their relative e-values with motif 1 having the best e-value. In subsequent discussions, the three motifs are referred to as motif A, motif B, and motif C, with motif A being furthest of the three from the C-terminus and motif C being the closest to the C-terminus. This naming system is used to allow meaningful comparison between results from different species where these motifs have different ranks of e-values relative to each other. The combined p-value of finding all of the identified motifs in a given odorant receptor peptide in the dataset ranged from 2.49e^-12^ to 2.62e^-38^. The combined p-value was the probability of finding a match of a sequence in the dataset to a group of motifs by random chance (Figure 2). Significantly, AgOr7, the mosquito ortholog of DmOr83b, has all three motifs at the C-terminal end (Figure 2, asterisk). When the number of motifs for MEME was increased to search for from three to eight motifs, only one additional motif was found with a significant distribution (2.9e^-295^, present in 63 of 79 AgOr peptides). This motif had limited sequence conservation with the notable exception of a histidine residue located approximately 70 residues to the N-terminal of *An. gambiae* motif A.

**Figure I.**
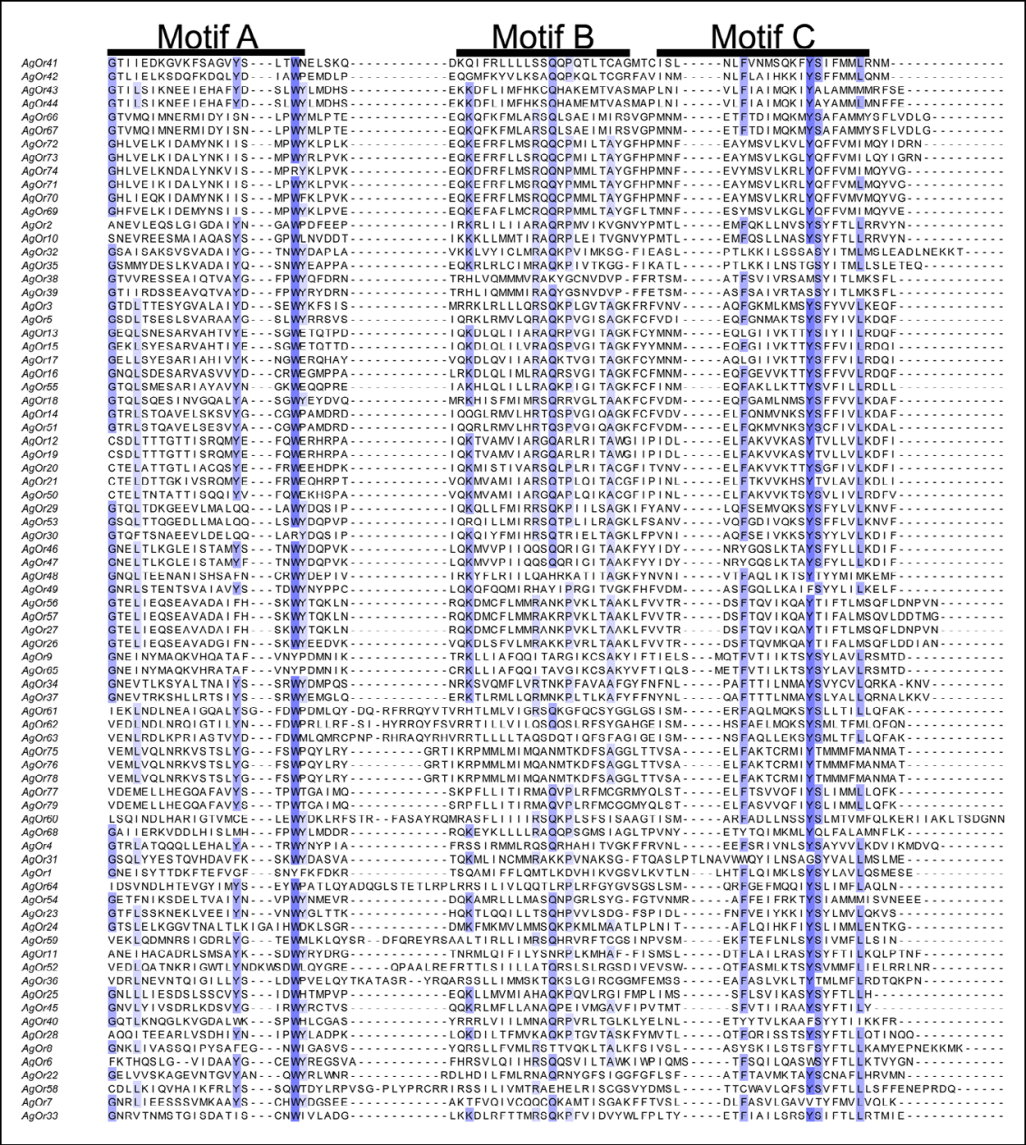
Multiple sequence alignment of the C-terminal region of all 79 *Anopheles gambiae* odorant receptors. AgOr peptides were aligned using ClustalW, and the subsequent alignment visualized using Jalview. A residue present at a given site in 50% or more of the AgOr peptides is boxed in blue. The more intense the blue the more often the residue is found at that site. Only the C-terminal region of the alignment is shown. The positions of motifs A, B, and C are shown. These motifs were not identified using alignment shown here. Instead they were identified using MEME. See Table 1 and Figure 3 for details.

Weblogo diagrams of motif A (Figure 3A), motif B (Figure 3B) and motif C (Figure 3C) illustrate the level of amino acid conservation within each motif at each position (Crooks et al. 2004). It is apparent from the weblogo diagrams that only a small portion of each motif consists of highly prevalent residues although there are additional areas where the chemical properties of the residues such as hydrophobicity, charge, and side chain structure are conserved. For example, in motif B of *An. gambiae* (Figure 3B) residue 1 and 2 are predominately positively charged residues while residues 4, 6, 7, and 8 are hydrophobic. The most highly conserved residues in the AgOr motifs are the tryptophan residue in motif A (Figure 3A), and a tyrosine/serine dyad in motif C (Figure 3C) mentioned above.

**Table I.**
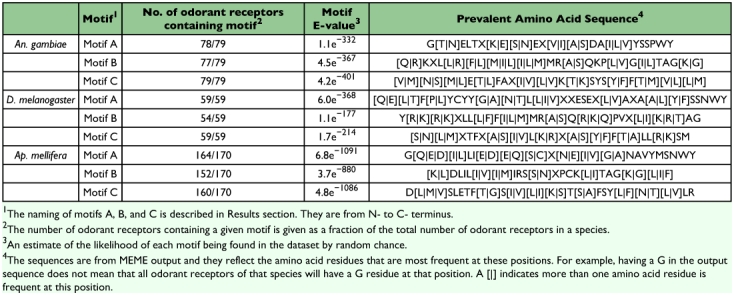
Three conserved C-terminal motifs in *An. gambiae*, *D. melanogaster*, and *Ap. mellifera* odorant receptor peptides.

### Odorant receptor c-terminal motifs are not found in gustatory receptors

Insect gustatory receptors are another family of putative G-protein coupled receptors. Insect gustatory receptors and odorant receptors are the closest relatives to each other in evolutionary terms (Clyne et al. 2000; Scott et al. 2001). DmGr21a in *D. melanogaster* is able to confer response to carbon dioxide (Suh et al. 2004) in conjunction with DmGr63a (Jones et al. 2007; Kwon et al. 2007). The motif alignment and search tool (MAST) (Bailey and Gribskov 1998) was used to search for the previously identified AgOr motifs in all 76 *An. gambiae* GRs (Hill et al. 2002). The best hit showed an e-value of 0.033 for a motif in the C-terminal region of one gustatory receptor. The poor e-value of the hit as well as further manual inspection suggests that it is not a true match. Thus this analysis indicates that the AgOr motifs are specific to odorant receptors and not a feature of G-protein coupled receptors.

**Figure 3.**
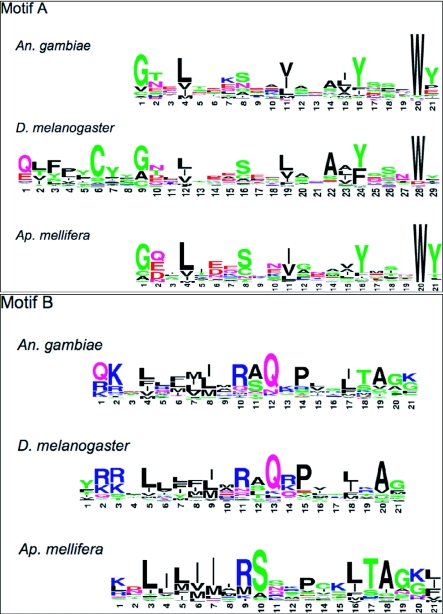
Weblogo presentation of motifs A, B, C in *Anophels gambiae*, *Drosophila melanogaster*, and *Apis mellirfera* odorant receptor peptides. Each line contains weblogo diagrams for motifs A, B, or C in one species. Weblogo diagrams indicate the prevalence of amino acids at specific positions. A). Weblogo presentation of motif A in all three species. B). Weblogo presentation of motif B in all three species.

**Figure 3 cont. C):**
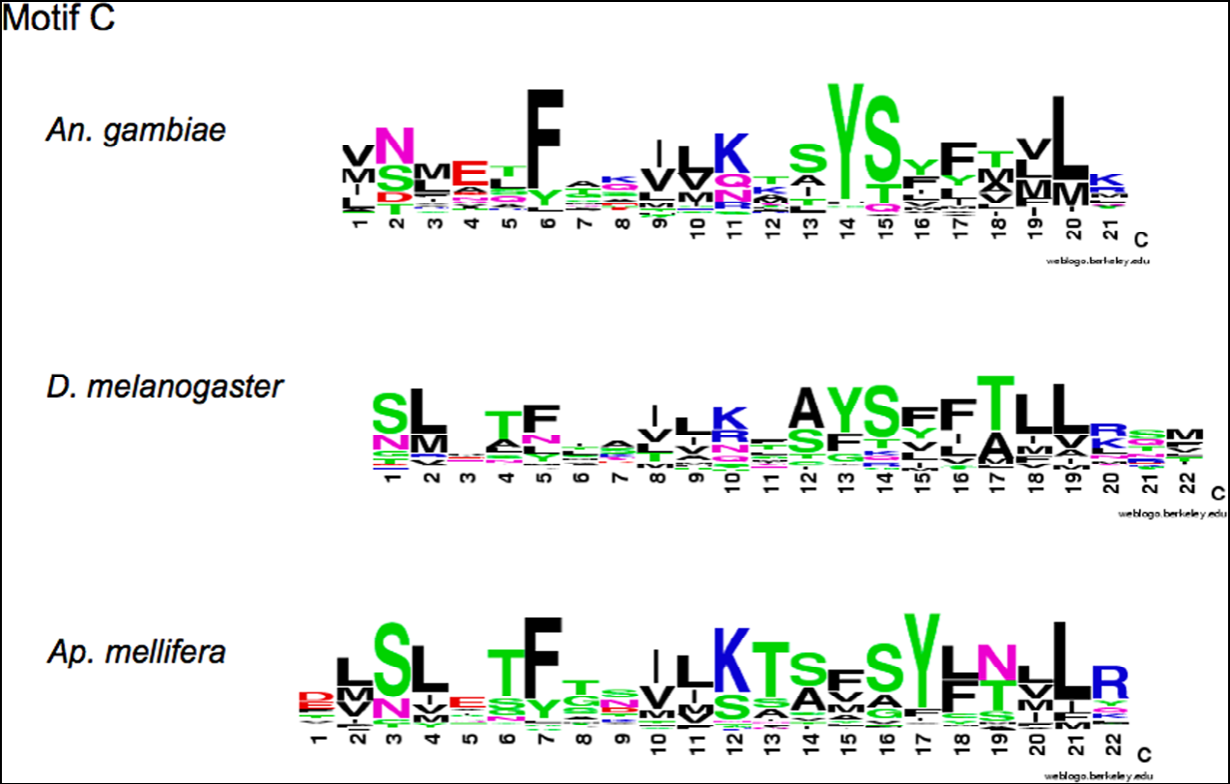
Weblogo presentation of motif C in all three species. Shown are weblogo diagrams indicating the prevalence of amino acids at specific positions in each motif.

### Odorant receptor c-terminal motifs are found in *D. melanogaster* and *Ap. mellifera* odorant receptors

A DmOr peptide database of 59 DmOrs was used as input into MEME to determine if any similar motifs existed in these odorant receptors. This analysis revealed three motifs found in the C-terminal end of a vast majority of these peptides (Table 1). All three motifs were found in 54 of 59 (92%) DmOrs. As was the case in AgOr peptides these three motifs are in the same order in all DmOr peptides. A side-by-side comparison of the weblogo diagrams from motifs A and B in both species reveals obvious similarities in sequence (Figure 3A and 3B). Most significant is the highly conserved tryptophan residue in motif A of both species. Part of motif A in DmOr peptides has been previously identified as the sequence of Phe-Pro-X-Cys-Tyr-(X)20-Trp (Vosshall 2003). The analysis showed several additionally conserved residues such as a glycine (residue 9) and a tyrosine/phenylalanine (residue 24). Motif C is very similar in both species in terms of their sequences and boundaries (Figure 3C).

Eight motifs were found in *Ap. mellifera* odorant receptor peptides (AmOr). Three of the motifs are apparent orthologs to the dipteran motifs A, B, and C (Table 1, Figure 3) both in terms of their sequence and relative location. Among the eight AgOr motifs, motifs A, B, and C ranked as number 1, 4, and 2 in terms of the significance of their respective e-values. The motif that had the third best e-value was near the middle of the receptor peptide, and is not shared with the dipteran receptors. Motifs ranked number 5 to 8 appear to have limited distribution in subgroups of AmOr peptides, and thus are not universal motifs in all AmOrs. These motifs are not further discussed in this paper. All three motifs are present in 147 of 170 (86%) AmOr peptides (Table 1, Figure 3). Motifs A, B, and C in AmOrs share similar sequence with dipteran Motifs A, B, and C respectively (Figure 3). For example there is a highly prevalent glycine residue followed by two variable residues, and then a highly prevalent leucine residue in motif A of all three species in addition to the conserved tryptophan residue. AmOr motif C is again very similar to the dipteran motifs (Figure 3C). However, instead of a tyrosine/serine dyad there is a phenylalanine/serine dyad in AmOr. The MEME analysis has therefore found three C-terminal motifs that are located in *An . gambiae*, *D. melanogaster* and *Ap. mellifera* odorant receptor peptides. Most of the residues in these motifs are not highly conserved, but several are highly prevalent across these diverse insect species.

## Discussion

Three motifs were located in the C-terminal ends of the odorant receptor peptides of three divergent insect species *An. gambiae*, *D. melanogaster*, and *Ap. mellifera* using a hidden markov model program. Table 1 lists the number of odorant receptors containing these motifs in each species, the e-value of the motifs, and the prevalent amino acid sequences of these motifs. The vast majority of insect odorant receptor peptides analyzed contain these C-terminal motifs. This is interesting considering that insect odorant receptor proteins are a very diverse family having very little conservation between species or within one species (Clyne et al. 1999; Vosshall et al. 1999; Robertson et al. 2003; Vosshall 2003). These motifs were not found in *An. gambiae* GRs despite the close evolutionary relationship between the odorant receptor and GR families (Clyne et al. 2000; Scott et al. 2001).

**Figure 4.**
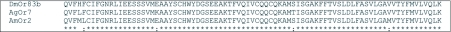
Conservation at the C-terminal regions of DmOr83b and its orthologs. Shown here is a ClustalW alignment of the last ∼90 amino acid residues of the Or83b family members in *Drosophila melanogaster* (DmOr83b), *Anopheles gambiae* (AgOr7), and *Apis mellifera*. (AmOr2). The relative position of motifs A, B, and C are shown.

Although all of the motifs described above had wide distribution in odorant receptors of the three species, motif B was not present in a small, but significant number of odorant receptors, especially in *Ap. mellifera* (Table 1). The absence of motif B may be explained by either technical or biological reasons, or both, as described below. Eleven of the 18 AmOrs lacking motif B had incomplete C-termini in current annotation, and two of the peptides were clearly pseudogenes (Robertson and Wanner 2006). Motif B was also not found in AmOr2, which is the honeybee ortholog of DmOr83b. However, a close inspection of the AmOr2 sequence revealed no amino acid substitution in the motif B region in comparison with DmOr83b and one substitution in comparison with AgOr7 (Figure 4). Therefore, sequence variation between motif B of the three species may explain why nearly identical sequences were recognized as motif B in DmOr83b and AgOr7 but not in AmOr2. Motif B was also lacking in two AgOrs and four DmOrs. Motif B was not as well conserved as the other two motifs (Figure 3). It is possible that the specific sequence of motif B is not as important as the chemical or structural properties of the residues in this motif. In comparing motif B in all three insect species (Figure 3B), some amino acid residues are present that are highly variable, but most of the residues in this region are hydrophobic in character. This conservation of hydrophobicity in these five residues may be functionally significant, while at the same time are difficult to be recognized by computer programs. It is also possible that motif B serves a role in enhancing a biological process, but is not absolutely required. For example, based on the working hypothesis that these C-terminal motifs are involved in protein-protein interactions, odorant receptor proteins lacking motif B might have a lower binding efficiency.

Having identified these motifs it is appropriate to ask why these motifs are present in the highly diverse insect odorant receptor family? As mentioned above, one possibility is that these motifs are involved in protein-protein inter-actions. There have been many efforts to identify protein-protein interaction sites through *in silico* methods, which resulted in the identification of several key characteristics. Protein-protein interaction sites are exposed on the surface of proteins and are hydrophobic, circular, and protruding (Janin and Chothia 1990; Young et al. 1994; Jones and Thornton 1995; Jones and Thornton 1997). Within these interaction areas are small “hot-spots” of a few residues contributing greatly to the overall binding energy of protein-protein interactions (Clackson and Wells 1995). In one survey it was found that tryptophan, tyrosine, and arginine are highly prevalent in these “hot-spots” (Bogan and Thorn 1998). Another study reported that tryptophan, phenylalanine, and methionine residues are significantly conserved in binding sites, but not on other exposed surfaces of proteins (Ma et al. 2003). Highly conserved and prevalent tryptophan, tyrosine, phenylalanine, and arginine residues were located in the C-terminal motifs of odorant receptors (Figure 3). We hypothesize that these motifs are protein-protein interaction sites, which would explain the conservation of only a few residues across the highly diverse insect odorant receptor protein family.

Unfortunately, at present there is no X-ray crystal structure of any insect odorant receptor or gustatory receptor that may illuminate the exact positioning of the newly discovered motifs and their potential role in protein-protein interaction. Hydrophobicity analysis can be useful at least in determining where residues are in relation to transmembrane helices. In this study the TMHMM server (http://www.cbs.dtu.dk/services/TMHMM/) was used to predict the transmembrane helices of five odorant receptors each from *An. gambiae*, *D. melanogaster*, and *Ap. mellifera* along with DmOr83b (data not shown). Motif A in all three species was found within helix 6, and perhaps part of the helix 6–7 loop. The difficulty of pinning down the exact positioning of helix 7 in particular makes this conclusion hard to draw unequivocally. It also makes further analysis of the positions of motif B and C uninformative except the supposition that these motifs lie near or in helix 7.

The vast majority of these insect odorant receptors maintained these motifs across hundreds of millions of years of evolution. This is impressive considering that the identity between insect odorant receptor peptides of the same species in some cases is as low as 17% (Vosshall 2003). These motifs have several highly conserved amino acids that were identified as being important in protein-protein interactions in other models. It is possible that these motifs allow odorant receptor-odorant receptor interactions as has been reported *in vitro* (Neuhaus et al. 2005). A more tantalizing prospect is that all or some of these motifs are involved in the formation of a heterodimer complex between DmOr83b or its ortholog and other odorant receptors (Neuhaus et al. 2005; Benton et al. 2006), a hypothesis that may be tested experimentally.

## Abbreviations

MEME -: multiple em for motif eliciation
MAST -: motif alignment and search tool
DmOr -: *Drosophila melanogaster* odorant receptor
AgOr -: *Anopheles gambiae* odorant receptor
AmOr -: *Apis mellifera* odorant receptor
